# Effectiveness of shortwave diathermy in patients with chronic low back pain: A study protocol for a randomised, single-blinded, multicentre clinical trial

**DOI:** 10.1371/journal.pone.0351060

**Published:** 2026-06-10

**Authors:** Mohammad Ali, Saddam Hossain, MD. Abu Bakar Siddiq, Monirul Islam

**Affiliations:** 1 Department of Physiotherapy and Rehabilitation, Uttara Adhunik Medical College and Hospital, Dhaka, Bangladesh; 2 Hasna Hena Pain Physiotherapy and Public Health Research Center, Dhaka, Bangladesh; 3 Department of Physiotherapy, International Institute of Health Sciences, Dhaka, Bangladesh; 4 Asia Digital Physiotherapy & Orthopedic Rehabilitation Center, Dhaka, Bangladesh; 5 Japan Bangladesh College of Physiotherapy and Health Sciences, Dhaka, Bangladesh; 6 Department of Physical Medicine and Rehabilitation, National Institute of Traumatology and Orthopaedic Rehabilitation, Dhaka, Bangladesh; Jashore University of Science and Technology (JUST), BANGLADESH

## Abstract

**Background:**

Chronic low back pain (CLBP) is a leading cause of disability worldwide and a major public health concern in low- and middle-income countries such as Bangladesh. Although international guidelines classify shortwave diathermy as a low-value treatment, it remains widely used in physiotherapy practice. Evidence on the effectiveness of continuous shortwave diathermy (CSWD) for CLBP is limited and inconsistent. This study aims to determine whether CSWD, when combined with standard physiotherapy, provides additional benefits compared with sham CSWD plus physiotherapy.

**Methods:**

This multicentre, randomised, single-blind, sham-controlled trial will recruit 208 participants aged 18–65 years with CLBP from tertiary hospitals and physiotherapy clinics in Dhaka, Bangladesh. Participants will be randomly allocated (1:1) to receive CSWD or sham CSWD, each combined with a standardised physiotherapy programme delivered three times weekly for four weeks. The intervention group will receive active CSWD (27.12 MHz, continuous mode, 20 min/session), while the control group will receive sham treatment. Primary outcomes include back pain intensity, leg pain intensity (0–10 numerical rating scale), and activity limitation (Oswestry Disability Index). Secondary outcomes include the Brief Pain Inventory, Global Rating of Change, Satisfaction with Treatment, DASS-21, Insomnia Severity Index, Treatment Credibility Questionnaire, and healthcare utilisation. Outcomes will be assessed at baseline, 4, 12, and 24 weeks. Data will be analysed using an intention-to-treat approach with linear mixed-effects models.

**Discussion:**

This trial will provide high-quality, context-specific evidence on the clinical and cost-effectiveness of CSWD for CLBP in a resource-constrained setting. The findings will inform physiotherapy practice in Bangladesh and may guide the appropriate use or de-implementation of this commonly used modality. The rigorous sham-controlled design will also contribute to the broader evidence base on electrotherapy for chronic pain.

## Introduction

Chronic low back pain (CLBP) is the leading cause of disability worldwide, imposing a substantial health, social, and economic burden on individuals and healthcare systems [1]. It affects over 600 million people globally and contributes significantly to work absenteeism, reduced productivity, and diminished quality of life [2]. In Bangladesh, community-based studies report a high prevalence of CLBP [3–6], while access to evidence-based rehabilitation remains limited [7]. Physiotherapy services are underutilised and often shaped by treatment paradigms that emphasise electrotherapy as a standalone intervention rather than integrating it into active, evidence-based approaches [8,9]. Although electrotherapy can be beneficial as an adjunct to rehabilitation, its isolated use may limit overall treatment effectiveness [10].

International clinical practice guidelines, including those from the National Institute for Health and Care Excellence and the American College of Physicians, generally classify shortwave diathermy (SWD) as a low-value treatment for non-specific low back pain due to inconsistent or limited evidence supporting its efficacy [11,12]. Despite this, SWD continues to be widely used in Bangladesh in both government and private physiotherapy settings [7,13,14]. In physician-led low back pain management pathways, physiotherapy is commonly recommended as part of the overall treatment strategy. The popularity of SWD among clinicians may be driven by traditional training curricula, patient preference for “heat-based” pain relief and limited local research evaluating its true clinical utility [14]. This discrepancy between international recommendations and local practice underscores the need for high-quality, context-specific evidence [10,15,16].

Shortwave diathermy is an electrotherapeutic modality that delivers high-frequency electromagnetic energy (27.12 MHz) to biological tissues, generating either thermal or non-thermal physiological effects depending on the treatment parameters. The proposed mechanisms include increased local circulation, reduction of muscle spasm, improved connective tissue extensibility, and facilitation of soft-tissue healing [17]. Experimental studies have demonstrated that SWD may also have effects such as modulation of inflammatory mediators, reduction of muscle hypertonicity, and enhancement of cellular repair processes [18]. However, the clinical relevance of these physiological effects in patients with CLBP remains uncertain [19].

Evidence from previous randomised controlled trials and systematic reviews on SWD for low back pain is inconsistent. Some studies have reported pain reduction and functional improvement with SWD compared to placebo or standard physiotherapy [20], while others found no clinically meaningful difference [21]. These conflicting results may be attributed to methodological limitations, including small sample sizes, absence of sham controls, heterogeneity in intervention protocols, and lack of follow-up beyond short-term outcomes. Consequently, guideline panels have continued to downgrade the recommendation for SWD use.

Furthermore, SWD devices are expensive and require substantial capital investment for clinics and hospitals. If the modality offers little or no added clinical benefit, such investment may not be economically justified, particularly in resource-constrained healthcare systems such as Bangladesh. Conversely, if evidence demonstrates that SWD provides meaningful pain relief or functional benefit as an adjunct to exercise-based physiotherapy, it may support its continued use under defined conditions.

To address these uncertainties, this multicentre, randomised, single-blinded, sham-controlled clinical trial aims to provide robust, context-specific evidence on the effectiveness of continuous SWD as an adjunct to standard physiotherapy in managing patients with chronic low back pain in Bangladesh.

## Objectives

**Primary objective:**

To determine the effectiveness of continuous SWD plus standard physiotherapy compared with sham SWD plus standard physiotherapy in reducing pain intensity and functional disability in patients with CLBP.

**Secondary objectives:**

- To compare the effects on functional disability, quality of life, mental health, sleep disorder and treatment credibility.
- To evaluate healthcare utilisation and participant global impression of change.

## Methods and analysis

This protocol has been developed in accordance with the SPIRIT 2013 statement and follows the reporting principles outlined in the CONSORT 2025 guidelines for interventional trials.

### Study design

This will be a multicentre, two-arm, parallel-group, randomised, single-blind (participant and assessor), sham-controlled clinical trial. The study aims to evaluate the effectiveness of continuous shortwave diathermy (CSWD) combined with standard physiotherapy compared with sham CSWD plus physiotherapy in adults with chronic low back pain (CLBP) (1:1). Participants and outcome assessors will be blinded to treatment allocation, while the treating physiotherapists cannot be blinded due to the perceptible heating sensation of active CSWD.

### Study setting

The trial will be conducted at 3 tertiary-level hospitals or physiotherapy centres in Dhaka, Bangladesh, under the supervision of a coordinating principal investigator. All sites will follow identical treatment and data-collection protocols to ensure standardisation.

### Eligibility criteria

#### Inclusion criteria.

- Age 18–65 years, either sex.
- Diagnosis of chronic nonspecific low back pain persisting ≥ 3 months.
- Pain located between the 12th rib and the inferior gluteal fold, with or without referred leg pain.
- Average baseline pain intensity ≥ 4 on the 0–10 numerical rating scale.
- Ability and willingness to give written informed consent.

#### Exclusion criteria.

- Red-flag conditions (fracture, tumour, infection, cauda equina syndrome).
- Prior spinal surgery or metallic implants/pacemakers.
- Pregnancy or malignancy.
- Ongoing structured physiotherapy or other investigational treatments for back pain.
- Cognitive impairment or inability to follow instructions.

### Interventions

All participants will receive a standardised physiotherapy programme administered by licensed physiotherapists three times per week for four weeks.

Sessions will include:

- Stretching of the hamstrings, piriformis, and lumbar paraspinals (3 × 30 s).
- Core-stabilisation training targeting transversus abdominis activation, bridging, and quadruped exercises (2 sets × 10 reps).
- Education on posture, ergonomics, and adherence to home exercises.

Participants will be encouraged to maintain normal activity levels but refrain from other physiotherapy or electrotherapy modalities during the study period.

#### Active CSWD (intervention group).

- Frequency: 27.12 MHz.
- Mode: Continuous wave.
- Power output: 120–180 W, adjusted for a pleasant warmth without discomfort.
- Application: Two plate-type electrodes applied bilaterally over the lumbar region using the coplanar technique, separated by 5–10 cm and insulated with a dry towel.
- Duration: 20 minutes per session, 3 sessions per week for 4 weeks.
- Device: The device appearance will be identical in both groups to maintain participant blinding.

#### Sham CSWD (control group).

Participants will be positioned identically with plates placed on the lumbar area for 20 minutes, but the device will remain off (0 W output).

They will be informed that the perception of warmth varies between individuals to maintain blinding credibility.

All treatments will be delivered by physiotherapists trained in CSWD application and safety. Adverse events or participant withdrawals will be documented at each visit.

### Outcome measures

#### Primary outcome measures.

1. Back-pain intensity: 0–10 numerical rating scale.
2. Leg-pain intensity: 0–10 numerical rating scale.
3. Activity limitation: Oswestry disability index.

#### Secondary outcome measures.

1. Brief Pain Inventory: pain severity and interference sub-scales.
2. Global Rating of Change: 7-point Likert scale.
3. Satisfaction with treatment: 5-point Likert scale.
4. Depression, Anxiety and Stress Scale (DASS-21).
5. Insomnia Severity Index (ISI).
6. Treatment Credibility Questionnaire.
7. Healthcare utilisation: participant diary recording consultations, imaging, and medication.

### Sample-size calculation

The sample size was calculated based on detecting a clinically meaningful between-group difference of 2 points on the 0–10 numerical rating scale for pain intensity, with an assumed standard deviation of 3, power of 80%, and a two-sided α of 0.05. The study is powered based on the primary outcome of pain intensity. This required 86 participants per group. Allowing for an anticipated attrition rate of 20%, the final target sample size is 208 participants (104 per group). The calculation was based on a single primary endpoint at the main follow-up time point. Although repeated measurements will be collected over time, the planned linear mixed-effects modelling approach will account for within-subject correlation and is expected to increase statistical efficiency.

### Randomisation and blinding

- Sequence generation: Computer-generated permuted block randomisation (block sizes of 4 and 6) stratified by centre.
- Allocation concealment: Sequentially numbered, opaque, sealed envelopes prepared by an independent researcher.
- Blinding: Participants and outcome assessors blinded; physiotherapists unblinded.
- Blinding assessment: At week 4, participants will guess group allocation to evaluate blinding success.

### Data collection and management

**Fig 1:**
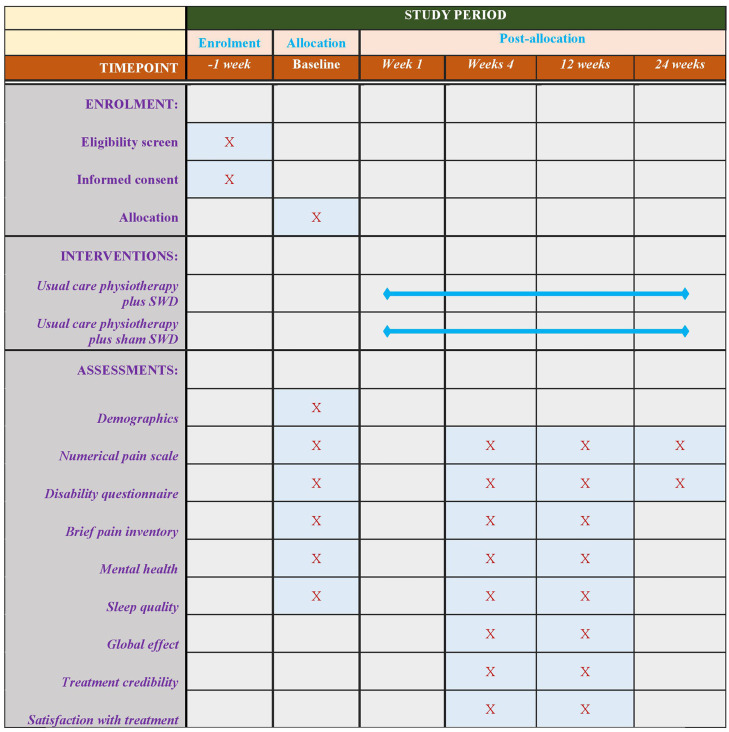
Schedule of enrollment, interventions, and assessments for study period based on SPIRIT guidelines.

**Fig 2:**
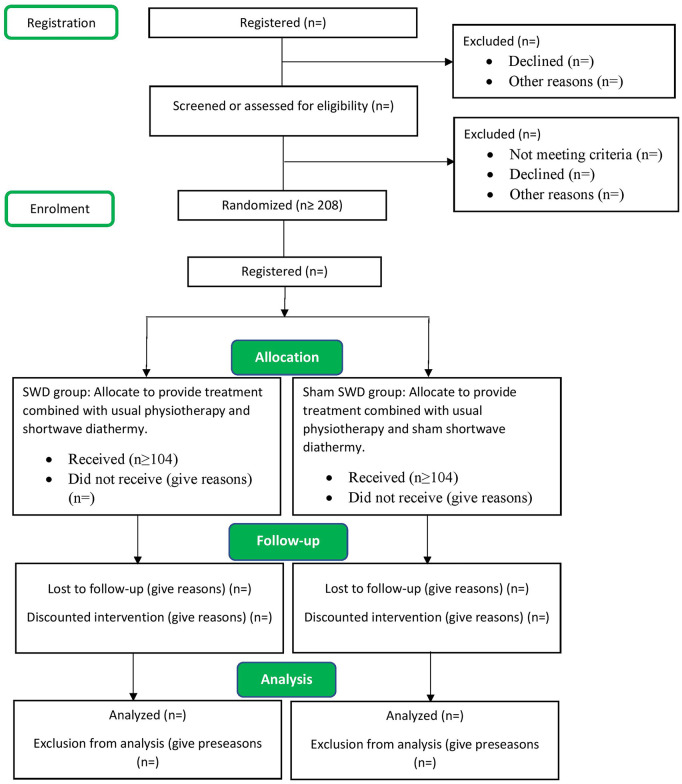
Study flow chart of enrolment, allocation, intervention, and assessment.

Data will be collected at baseline, 4^th^, 12^th^ and 24^th^ week post treatment (Fig 1). Data will be recorded in paper-based questionnaires and transferred to Microsoft Excel before being entered into a password-protected database. Quality-control measures include double-data entry, random verification of 10% of records, and automated range checks. All identifiable information will be stored separately on encrypted drives with restricted access. Furthermore, Fig 2 presents the flow of participants throughout the study.

### Statistical analysis

All analyses will follow the intention-to-treat principle.

- Primary outcomes: Changes in numerical rating scale and Oswestry disability index scores over time will be analysed using linear mixed-effects models (group, time, and interaction as fixed effects; participants as random effects).
- Secondary outcomes: Continuous variables analysed similarly; categorical variables by logistic regression or χ² tests.
- Post-hoc testing: Bonferroni correction for multiple comparisons.
- Missing data: Addressed by multiple imputation via chained equations (MICE) under the assumption that data are missing at random. The imputation model will include baseline outcomes, treatment allocation, and relevant demographic variables. At least 20 imputations will be generated.

To assess potential attrition bias, baseline characteristics of participants who complete the study and those who do not will be compared. In addition to the intention-to-treat analysis, a per-protocol analysis including participants completing ≥80% of treatment sessions will be conducted.

All analyses will be conducted using SPSS v29.0 (IBM, USA)**,** with significance set at p < 0.05.

### Intervention standardization and monitoring

All physiotherapists will undergo standardized training using a treatment manual. Intervention fidelity will be ensured through monitoring and checklists. A trial-steering committee will supervise protocol adherence and data integrity. Given the non-invasive nature of CSWD, a formal Data Safety Monitoring Board is not planned. All adverse events and serious adverse events will be reported to the ethics committee within 24 hours.

### Ethics and dissemination

Ethical approval was obtained from the Ethical Review Committee of Uttara Adhunik Medical College (UAMC/IRB/ERC/Recommend- 01/2025, date 30/09/2025). The trial was registered with the Clinical Trials Registry – India (CTRI) under (CTRI/2025/11/097194; registered 11 November2025). Results will be submitted for publication in peer-reviewed journals and presented at national and international conferences. Participants will receive a plain-language summary of findings.

### Patient and public involvement

Patients with CLBP were not consulted during the development of the trial schedule and assessment selection to ensure feasibility and relevance. They will not be involved in reviewing and disseminating study results.

### Trial status and timeline

This is the second version (2.0) of the study protocol, dated May 2026. The timeline for the study is as follows:

- Participant Recruitment: Recruitment is anticipated to begin in June 2026 and is expected to be completed by June 2027.
- Data Collection: Data collection for the final follow-up assessment (24-week) is projected to be completed by December 2027.
- Data Analysis and Results: Data cleaning, analysis, and interpretation are scheduled to commence after the final follow-up. The results of the primary analysis are expected to be available by June 2028.
- Based on patient flow (10–15 CLBP patients/week per centre), recruitment of 208 participants across three centres is feasible within the planned timeframe.

As of the submission of this protocol, no participants have been recruited, enrolled, or allocated to study interventions. No data collection has taken place.

## Discussion

This trial aims to determine whether adding CSWD to standard physiotherapy offers additional benefits in pain reduction, disability, and psychosocial outcomes among individuals with CLBP. Although SWD has been traditionally used in physiotherapy practice, its clinical value remains uncertain. Recent evidence, including Amaral et al. (2023), reported no significant advantage of CSWD when combined with exercise therapy, suggesting that the improvements observed may be primarily attributed to active rehabilitation rather than heat application [21].

However, in Bangladesh, SWD continues to be widely used in both government and private physiotherapy settings despite limited high-quality evidence [14]. This study is designed to fill that evidence gap through a well-controlled, sham-blinded, multicentre randomised trial. The use of validated outcome measures such as the Oswestry Disability Index, Brief Pain Inventory, and DASS-21 will allow a comprehensive evaluation of both physical and psychological effects.

Potential limitations include the inability to blind therapists, variation in adherence to the home exercise program, and relatively short treatment duration. Nonetheless, the study’s rigorous design, adequate sample size, and inclusion of a sham control are expected to provide strong, locally relevant evidence regarding the true effectiveness of CSWD.

## Conclusion

This study will clarify whether CSWD adds any measurable benefit to physiotherapy in managing CLBP. The findings are expected to guide evidence-based physiotherapy practice in Bangladesh and contribute to international understanding of heat-based modalities in chronic pain management.

## Supporting information

S1 File SPIRIT 2025 Checklist – Filled for PLOS ONE Submission. (DOCX)

S2 File Full_Study_Protocol_IRB_PLOS. (DOCX)

## Abbreviations

AE: Adverse Event
CLBP: Chronic Low Back Pain
CRF: Case Report Form
CSI-9: Central Sensitisation Inventory-9
ITT: Intention-to-Treat
PGIC: Patient Global Impression of Change
RMDQ: Roland-Morris Disability Questionnaire
SAE: Serious Adverse Event
SPIRIT: Standard Protocol Items: Recommendations for Interventional Trials
SWD: Shortwave Diathermy
VAS: Visual Analogue Scale
