# Supplementary material for: Effectiveness of shortwave diathermy in patients with chronic low back pain: A study protocol for a randomised, single-blinded, multicentre clinical trial
Source: PLoS One. 2026 Jun 10;21(6):e0351060. doi: 10.1371/journal.pone.0351060 (PMC13252720; doi:10.1371/journal.pone.0351060)
Supplement: S1 File — (DOCX) [file pone.0351060.s001.docx]

# SPIRIT 2025 Checklist – Filled for PLOS ONE Submission

| Item No. | Description | Reported Page No. |
| --- | --- | --- |
| 1a | Title stating the trial design, population, and interventions | Page 1 |
| 1b | Structured summary | Pages 3–4 |
| 2 | Protocol version | Page 13–14 |
| 3a | Names, affiliations, and roles of protocol contributors | Pages 1–2 |
| 3b | Trial sponsor contact information | Page 2 |
| 3c | Role of sponsor/funders | Page 16 |
| 3d | Trial oversight committees | Page 12–13 |
| 4 | Trial registration | Page 4 |
| 5 | Where protocol & SAP can be accessed | Page 15 |
| 6 | Data sharing statement | Page 15 |
| 7a | Funding sources | Page 16 |
| 7b | Conflicts of interest | Page 15 |
| 8 | Dissemination policy | Page 13 |
| 9a | Scientific background and rationale | Pages 5–7 |
| 9b | Explanation for choice of comparator | Pages 6–7 |
| 10 | Objectives | Page 7 |
| 11 | Patient and public involvement | Page 13 |
| 12 | Trial design | Pages 7–8 |
| 13 | Trial setting | Page 8 |
| 14a | Eligibility criteria for participants | Page 8 |
| 14b | Eligibility criteria for sites/personnel | Page 8 |
| 15a | Interventions & comparator description | Pages 9–10 |
| 15b | Criteria for discontinuation/modification | Page 10 |
| 15c | Adherence strategies | Page 11 |
| 15d | Concomitant care permitted/prohibited | Page 9 |
| 16 | Primary & secondary outcomes | Pages 10–11 |
| 17 | Harms | Page 12 |
| 18 | Participant timeline | Figures on Page 19 |
| 19 | Sample size calculation | Page 11 |
| 20 | Recruitment plan | Page 14 |
| 21a | Sequence generation | Page 11 |
| 21b | Type of randomisation/stratification | Page 11 |
| 22 | Allocation concealment mechanism | Page 11 |
| 23 | Implementation of randomisation | Page 11 |
| 24a | Blinding – who is blinded | Page 11 |
| 24b | Blinding procedures | Page 11 |
| 24c | Unblinding criteria | Not stated |
| 25a | Data collection methods | Pages 11–12 |
| 25b | Retention/complete follow-up plans | Page 12 |
| 26 | Data management | Page 12 |
| 27a | Statistical methods | Page 12 |
| 27b | Analysis population | Page 12 |
| 27c | Missing data handling | Page 12 |
| 27d | Additional analyses | Page 12 |
| 28a | Data Monitoring Committee | Page 12–13 |
| 28b | Interim analyses | Not applicable (no DSMB) |
| 29 | Trial monitoring | Page 12–13 |
| 30 | Research ethics approval | Page 13 |
| 31 | Protocol amendments plan | Page 13 |
| 32a | Informed consent procedures | Page 15 |
| 32b | Additional consent for specimens | Not applicable |
| 33 | Confidentiality | Page 12 |
| 34 | Ancillary/post-trial care | Not applicable |
