# Supplementary material for: Effectiveness of shortwave diathermy in patients with chronic low back pain: A study protocol for a randomised, single-blinded, multicentre clinical trial
Source: PLoS One. 2026 Jun 10;21(6):e0351060. doi: 10.1371/journal.pone.0351060 (PMC13252720; doi:10.1371/journal.pone.0351060)
Supplement: S2 File — (DOCX) [file pone.0351060.s002.docx]

**S4 File. Full study protocol submitted for ethical review**

**Effectiveness of Continuous Shortwave Diathermy in Patients with Chronic Low Back Pain: A Randomised, Single-Blinded, Multicentre Clinical Trial**

*Short title: Effectiveness of continuous shortwave diathermy for chronic low back pain: a protocol for a randomised controlled trial*

## Principal investigator

Mohammad Ali
Department of Physiotherapy and Rehabilitation
Uttara Adhunik Medical College and Hospital
Dhaka, Bangladesh

## Co-investigators

Saddam Hossain
MD. Abu Bakar Siddiq
Monirul Islam

## Protocol information

| **Item** | **Details** |
| --- | --- |
| Protocol version | Version 1.0 |
| Protocol date | 30 September 2025 |
| Ethics approval committee | Ethical Review Committee of Uttara Adhunik Medical College |
| Ethics reference number | UAMC/IRB/ERC/Recommend-01/2025 |
| Ethics approval date | 30 September 2025 |
| Trial registration | Clinical Trials Registry-India, CTRI/2025/11/097194; registered 11 November 2025 |

This document presents the full study protocol submitted for ethical review and approved by the Ethical Review Committee of Uttara Adhunik Medical College. It has been prepared as a clean Supporting Information file for journal submission, with no signatures, logos, stamps, confidentiality statements, identifying images, or copyrighted images/content included.

# 1. Background and rationale

Chronic low back pain (CLBP) is the leading cause of disability worldwide and imposes a substantial health, social, and economic burden on individuals and healthcare systems. It affects more than 600 million people globally and contributes substantially to work absenteeism, reduced productivity, and reduced quality of life.

In Bangladesh, access to evidence-based rehabilitation remains limited despite the high burden of low back pain. Physiotherapy services are underutilised and are often shaped by treatment paradigms that emphasise electrotherapy as a standalone intervention rather than integrating it into active, evidence-based approaches. Although electrotherapy can be beneficial as an adjunct to rehabilitation, its isolated use may limit overall treatment effectiveness.

Shortwave diathermy (SWD) is widely used in physiotherapy practice in Bangladesh despite international guidelines generally classifying SWD as a low-value treatment for nonspecific low back pain because of inconsistent evidence regarding its effectiveness. In physician-led low back pain management pathways, physiotherapy is commonly recommended as part of the overall treatment strategy. The popularity of SWD among clinicians may be influenced by traditional training curricula, patient preference for heat-based pain relief, and limited local research evaluating its true clinical value.

Continuous shortwave diathermy (CSWD) delivers high-frequency electromagnetic energy to biological tissues and may produce thermal and physiological effects, including increased local circulation, reduced muscle spasm, improved connective tissue extensibility, and facilitation of tissue healing. However, the clinical relevance of these physiological effects in patients with CLBP remains uncertain.

Previous randomised controlled trials investigating SWD for low back pain have shown inconsistent results. Some studies have reported reductions in pain and improvements in function, while others have found no clinically meaningful benefit. These conflicting findings may be explained by small sample sizes, lack of sham control groups, variation in intervention protocols, and short follow-up periods.

Given the frequent use of SWD in Bangladesh and the uncertainty regarding its clinical effectiveness, a rigorous sham-controlled randomised trial is needed. This study aims to generate context-specific evidence regarding whether CSWD provides additional benefit when used as an adjunct to standard physiotherapy for patients with CLBP.

# 2. Objectives

## 2.1 Primary objective

To determine whether continuous shortwave diathermy combined with standard physiotherapy is more effective than sham shortwave diathermy combined with standard physiotherapy in reducing pain intensity and disability in patients with chronic low back pain.

## 2.2 Secondary objectives

1. To compare the effects of active CSWD and sham CSWD on psychosocial outcomes and sleep disturbance.
2. To evaluate treatment credibility and participant satisfaction.
3. To assess healthcare utilisation and participant global impression of change.
4. To determine whether CSWD provides clinically useful additional benefit as an adjunct to standard physiotherapy in a resource-constrained setting.

# 3. Study design

This study is a multicentre, two-arm, parallel-group, randomised, single-blind, sham-controlled clinical trial.

Participants will be randomly allocated in a 1:1 ratio to one of two groups: active CSWD plus standard physiotherapy, or sham CSWD plus standard physiotherapy.

Participants and outcome assessors will be blinded to treatment allocation. Treating physiotherapists cannot be blinded because active CSWD produces perceptible warmth.

Outcome assessments will be conducted at baseline, week 4, week 12, and week 24. The total follow-up period will be 24 weeks.

# 4. Study setting

The trial will be conducted at three tertiary-level hospitals or physiotherapy centres in Dhaka, Bangladesh:

1. Uttara Adhunik Medical College and Hospital
2. Asia Digital Physiotherapy & Orthopedic Rehabilitation Center
3. Japan Bangladesh College of Physiotherapy and Health Sciences

All participating centres will follow identical treatment, assessment, data-collection, and safety-monitoring procedures.

# 5. Recruitment period and study timeline

Recruitment is planned to begin in June 2026 and is expected to be completed by June 2027. The final 24-week follow-up assessment is expected to be completed by December 2027. Data cleaning, analysis, and interpretation are expected to be completed by June 2028.

Based on patient flow at participating centres, each centre is expected to identify approximately 10-15 potentially eligible patients with CLBP per week. Recruitment of 208 participants across three centres is therefore considered feasible within the planned recruitment period.

No participants have been enrolled at the time of protocol submission.

# 6. Participant characteristics

Participants will be adults aged 18-65 years with chronic nonspecific low back pain lasting at least three months. Participants may have low back pain with or without referred leg pain and must report an average baseline pain intensity of at least 4 on a 0-10 numerical rating scale. Both male and female participants will be eligible.

# 7. Eligibility criteria

## 7.1 Inclusion criteria

1. Age 18-65 years.
2. Male or female.
3. Diagnosis of chronic nonspecific low back pain persisting for at least three months.
4. Pain located between the 12th rib and inferior gluteal fold, with or without referred leg pain.
5. Average baseline pain intensity of at least 4 on a 0-10 numerical rating scale.
6. Ability and willingness to provide written informed consent.
7. Ability to attend treatment sessions and follow-up assessments.

## 7.2 Exclusion criteria

1. Red-flag conditions, including suspected fracture, tumour, infection, or cauda equina syndrome.
2. Previous spinal surgery.
3. Metallic implants or pacemaker.
4. Pregnancy.
5. Malignancy.
6. Ongoing structured physiotherapy or other investigational treatments for low back pain.
7. Cognitive impairment or inability to follow instructions.
8. Any contraindication to shortwave diathermy.

# 8. Screening and informed consent

Potential participants will be identified from outpatient physiotherapy and rehabilitation services at participating centres. Trained research staff will screen potential participants using the predefined eligibility criteria.

Eligible participants will receive verbal and written information about the study, including its purpose, procedures, potential benefits, potential risks, voluntary nature of participation, confidentiality, and right to withdraw at any time without penalty.

Written informed consent will be obtained before any study-specific procedure, baseline assessment, or randomisation is performed.

# 9. Randomisation and allocation concealment

Randomisation will be conducted using a computer-generated random allocation sequence. Permuted block randomisation with block sizes of 4 and 6 will be used and stratified by centre.

Allocation concealment will be maintained using sequentially numbered, opaque, sealed envelopes prepared by an independent researcher who is not involved in participant recruitment, treatment delivery, or outcome assessment.

After baseline assessment and informed consent, the treating physiotherapist will open the next sequential envelope to determine group allocation.

# 10. Blinding

Participants and outcome assessors will be blinded to treatment allocation. Treating physiotherapists cannot be blinded because active CSWD produces a perceptible heating sensation.

To maintain participant blinding, both groups will receive identical positioning and device placement. Device appearance will be identical in both groups where possible. Participants in both groups will be informed that the perception of warmth varies between individuals.

At week 4, participants will be asked to guess their group allocation to assess blinding success.

# 11. Interventions

All participants will receive a standardised physiotherapy programme three times per week for four weeks. The only difference between groups will be whether CSWD is active or sham.

## 11.1 Standard physiotherapy programme for both groups

The standard physiotherapy programme will be delivered by licensed physiotherapists and will include stretching exercises, core-stabilisation exercises, postural education, and home exercise advice.

Each treatment session will include stretching of hamstrings, piriformis, and lumbar paraspinal muscles. Each stretch will be performed as three repetitions of 30 seconds. Core-stabilisation exercises will include transversus abdominis activation, bridging, and quadruped exercises, performed as two sets of 10 repetitions according to participant tolerance.

Participants will receive education on posture, ergonomics, maintaining usual activity, and adherence to home exercises. Participants will be encouraged to maintain normal activity levels but will be advised not to receive other physiotherapy or electrotherapy treatments for low back pain during the intervention period.

## 11.2 Active CSWD group

Participants allocated to the active CSWD group will receive continuous shortwave diathermy in addition to standard physiotherapy.

- Frequency: 27.12 MHz.
- Mode: continuous wave.
- Power output: 120-180 W.
- Intensity: adjusted to produce comfortable warmth without pain or discomfort.
- Duration: 20 minutes per session.
- Treatment frequency: three sessions per week for four weeks.
- Application method: two plate-type electrodes applied bilaterally over the lumbar region using the coplanar technique.
- Electrode separation: approximately 5-10 cm.
- Insulation: dry towel insulation will be used.

## 11.3 Sham CSWD group

Participants allocated to the sham CSWD group will receive sham CSWD in addition to standard physiotherapy. Participants will be positioned identically to the active CSWD group. Plate electrodes will be placed over the lumbar region for 20 minutes; however, no energy output will be delivered, and the device will remain at 0 W. Participants will be informed that warmth perception varies between individuals to support blinding credibility.

# 12. Intervention standardisation and adherence

All physiotherapists involved in treatment delivery will receive structured training before study initiation using a standardised treatment manual. The training will include CSWD safety procedures, electrode placement, sham procedures, exercise delivery, participant education, and adverse event documentation.

Treatment fidelity will be monitored using standardised treatment checklists, periodic site supervision, and trial steering committee oversight.

Attendance will be recorded at each treatment session. Participants who complete at least 80% of scheduled treatment sessions will be considered adherent for per-protocol analysis.

# 13. Outcome measures

## 13.1 Primary outcomes

1. Back pain intensity measured using a 0-10 numerical rating scale.
2. Leg pain intensity measured using a 0-10 numerical rating scale.
3. Activity limitation measured using the Oswestry Disability Index.

## 13.2 Secondary outcomes

1. Brief Pain Inventory, including pain severity and interference subscales.
2. Global Rating of Change.
3. Satisfaction with Treatment.
4. Depression, Anxiety and Stress Scale-21.
5. Insomnia Severity Index.
6. Treatment Credibility Questionnaire.
7. Healthcare utilisation diary, including consultations, imaging, and medication use.

# 14. Assessment schedule

Outcome assessments will be conducted by trained assessors blinded to treatment allocation. Assessments will take place at baseline, week 4, week 12, and week 24. Primary and secondary outcomes will be collected at each assessment time point where applicable.

| Outcome | Baseline | Week 4 | Week 12 | Week 24 |
| --- | --- | --- | --- | --- |
| Pain intensity | Yes | Yes | Yes | Yes |
| Oswestry Disability Index | Yes | Yes | Yes | Yes |
| Secondary outcomes | Yes | Yes | Yes | Yes |

# 15. Data collection and management

Data will be collected using paper-based questionnaires and transferred to a password-protected electronic database.

Data quality-control procedures will include double data entry, random verification of records, automated range checks, and review of missing or inconsistent data.

Each participant will be assigned a unique study identification number. Identifiable information will be stored separately from study data on encrypted or password-protected storage with restricted access. Only authorised study personnel will have access to identifiable participant information.

# 16. Sample size calculation

The sample size was calculated based on detecting a clinically meaningful between-group difference of 2 points on the 0-10 numerical rating scale for pain intensity. The assumptions were: standard deviation of 3, power of 80%, and two-sided alpha of 0.05.

This required 86 participants per group. Allowing for 20% attrition, the final target sample size is 208 participants, with 104 participants per group.

The study is powered based on the primary outcome of pain intensity. Although repeated measurements will be collected over time, the planned linear mixed-effects modelling approach will account for within-subject correlation and is expected to increase statistical efficiency.

# 17. Statistical analysis

All analyses will follow the intention-to-treat principle.

Primary outcomes will be analysed using linear mixed-effects models. The models will include group, time, and group-by-time interaction as fixed effects, and participant as a random effect.

Continuous secondary outcomes will be analysed using similar mixed-effects models where appropriate. Categorical outcomes will be analysed using logistic regression or chi-square tests. Post-hoc comparisons will be adjusted using Bonferroni correction where appropriate.

Missing data will be handled using Multiple Imputation by Chained Equations under the assumption that data are missing at random. The imputation model will include baseline outcomes, treatment allocation, and relevant demographic variables. At least 20 imputations will be generated.

To assess potential attrition bias, baseline characteristics of participants who complete the study and those who do not will be compared.

A per-protocol sensitivity analysis will include participants who complete at least 80% of treatment sessions. All statistical analyses will be conducted using SPSS version 29.0 or later. Statistical significance will be set at p < 0.05.

# 18. Safety monitoring and adverse event reporting

CSWD is a non-invasive intervention and is expected to involve minimal risk. Potential adverse effects include mild warmth, temporary discomfort, or transient symptom aggravation.

An adverse event will be defined as any undesirable experience occurring during the trial, whether or not considered related to the intervention. A serious adverse event will include any event resulting in death, life-threatening illness, hospitalisation, significant disability, or any medically important event.

All adverse events will be recorded at each treatment visit and follow-up assessment. Serious adverse events will be reported to the Ethical Review Committee within 24 hours.

A trial steering committee will supervise protocol adherence, safety monitoring, and data integrity. Given the non-invasive nature of CSWD, a formal Data Safety Monitoring Board is not planned.

# 19. Ethical considerations

Ethical approval has been obtained from the Ethical Review Committee of Uttara Adhunik Medical College.

Ethics reference number: UAMC/IRB/ERC/Recommend-01/2025. Approval date: 30 September 2025.

Written informed consent will be obtained from all participants before any study procedures are undertaken. Participation will be voluntary. Participants may withdraw at any time without penalty or effect on their usual care.

The trial is registered with the Clinical Trials Registry-India. Trial registration number: CTRI/2025/11/097194. Registration date: 11 November 2025.

# 20. Confidentiality

Participant confidentiality will be maintained throughout the study.

All personal information will be stored securely and separately from study data. Study data will be identified using coded participant numbers rather than names or other direct identifiers.

Only deidentified, aggregated findings will be reported in publications and presentations.

# 21. Data sharing

Deidentified participant data generated during the study will be made publicly available in an open-access repository upon completion of the study and publication of the primary findings.

# 22. Funding and competing interests

This study has received no specific funding. The investigators declare no competing interests.

# 23. Dissemination plan

The findings of the study will be submitted for publication in peer-reviewed journals and presented at national and international scientific conferences. A plain-language summary of the findings will be provided to participants and participating centres where feasible.

# 24. References

1. Buchbinder R, van Tulder M, Oeberg B, Costa LM, Woolf A, Schoene M, et al. Low back pain: a call for action. The Lancet. 2018;391:2384-2388. https://doi.org/10.1016/S0140-6736(18)30488-4.

2. Li Y, Zou C, Guo W, Han F, Fan T, Zang L, et al. Global burden of low back pain and its attributable risk factors from 1990 to 2021: a comprehensive analysis from the Global Burden of Disease Study 2021. Front Public Health. 2024;12:1480779. https://doi.org/10.3389/FPUBH.2024.1480779.

3. Ali M, Bonna AS, Sarkar A, Islam MdA, Rahman N-A-S. SARS-CoV-2 infection is associated with low back pain: findings from a community-based case-control study. International Journal of Infectious Diseases. 2022;122:144-151. https://doi.org/10.1016/j.ijid.2022.05.050.

4. Ali M, Hossain A, Ahsan GU. Prevalence and associated occupational factors for low back pain among the bank employees in Dhaka City. MedRxiv. 2019:19012328. https://doi.org/10.1101/19012328.

5. Ali M, Islam M, Abu Bakar Siddiq Md, Khan Pranto N, Akter M, Akter Munny M, et al. Exploring the impact of occupational factors on low back pain in ride-sharing motorbike drivers in Bangladesh: A comprehensive cross-sectional analysis. Prev Med Rep. 2024;43:102788. https://doi.org/10.1016/j.pmedr.2024.102788.

6. Ali M, Siddiq MdAB, Pranto NK, Amran NH, Akter M, Munny MA, et al. Prevalence and predictors of musculoskeletal health complaints among sedentary, monotonous urban workers: A survey in Bangladesh. PLoS One. 2023;18:e0282922. https://doi.org/10.1371/journal.pone.0282922.

7. Ali M, Uddin Z, Hossain A. Clinical practice pattern of managing low back pain among physiotherapists in Bangladesh: A cross-sectional study. Physiother Pract Res. 2022;43:275-282. https://doi.org/10.3233/PPR-210549.

8. Ali M, Ford JJ, Hossain A, Hahne AJ. Transforming chronic low back pain management in Bangladesh: implications for rehabilitation in low- and middle-income countries. Annals of Medicine & Surgery. 2026. https://doi.org/10.1097/MS9.0000000000004977.

9. Ali M, Peiris CL, Ford JJ, Hossain A, Hahne AJ. Perceptions of Low Back Pain and the Role of Physiotherapy in Bangladesh: A Qualitative Study. Musculoskeletal Care. 2025;23:e70159. https://doi.org/10.1002/MSC.70159.

10. Ali M, Miller T. Continuous Passive Motion in the Rehabilitation of a Comminuted Patellar Fracture Following Delayed Physiotherapy Initiation: A Case Report. Clin Case Rep. 2026;14:e71806. https://doi.org/10.1002/CCR3.71806.

11. NICE. Recommendations: Low back pain and sciatica in over 16s: assessment and management. NICE; 2016.

12. Qaseem A, Wilt TJ, McLean RM, Forciea MA. Noninvasive treatments for acute, subacute, and chronic low back pain: a clinical practice guideline from the American College of Physicians. Ann Intern Med. 2017;166:514-530. https://doi.org/10.7326/M16-2367.

13. Ahmed M, Hoque A, Zannat NA, Sadeque ABMZ, Khan TI. Comparison of the efficacy of Transcutaneous Electrical Nerve Stimulation and Short Wave Diathermy on patients with chronic nonspecific low back pain. TAJ: Journal of Teachers Association. 2023;36:135-143. https://doi.org/10.3329/TAJ.V36I2.72492.

14. Ahmed MS, Shakoor MA, Khan AA. Evaluation of the effects of shortwave diathermy in patients with chronic low back pain. Bangladesh Med Res Counc Bull. 2009;35:18-20. https://doi.org/10.3329/BMRCB.V35I1.2320.

15. Ali M, Ford JJ, Hossain A, Danazumi MS, Hahne AJ. Implementing individualised physiotherapy using the Specific Treatment of Problems of the Spine approach for chronic low back pain in Bangladesh: Protocol for a prospective sequential comparison clinical trial. Contemp Clin Trials. 2025;154. https://doi.org/10.1016/j.cct.2025.107960.

16. Ali M, Hahne A, Ford J. Individualised physiotherapy using the Specific Treatment of Problems of the Spine approach is more effective than usual physiotherapy care for chronic low back pain in Bangladesh: results from a prospective sequential comparison clinical trial. Osteoarthritis Cartilage. 2026;34:S100. https://doi.org/10.1016/J.JOCA.2026.01.112.

17. Lehmann JF, Warren CG, Scham SM. Therapeutic heat and cold. Clin Orthop Relat Res. 1974;99:207-245. https://doi.org/10.1097/00003086-197403000-00028.

18. Fu T, Lineaweaver WC, Zhang F, Zhang J. Role of shortwave and microwave diathermy in peripheral neuropathy. Journal of International Medical Research. 2019;47:3569-3579. https://doi.org/10.1177/0300060519854905.

19. Shields N, Gormley J, O Hare N. Short-wave diathermy: current clinical and safety practices. Physiother Res Int. 2002;7:191-202. https://doi.org/10.1002/PRI.259.

20. Shakoor MA, Al Hasan S, Moyeenuzzaman M, Deb AK. Treatment with Short Wave Diathermy on chronic low back pain. Journal of Chittagong Medical College Teachers Association. 2010;21:40-44. https://doi.org/10.3329/jcmcta.v21i1.7669.

21. Amaral S, Passaro AC, Casarotto RA. Effect of the association of continuous shortwave diathermy and Pilates-based exercises on pain, depression, and anxiety in chronic non-specific low back pain: a randomized clinical trial. Brazilian Journal of Medical and Biological Research. 2023;56:e12338. https://doi.org/10.1590/1414-431X2023E12338.
