# Supplementary material for: Effectiveness of shortwave diathermy in patients with chronic low back pain: A study protocol for a randomised, single-blinded, multicentre clinical trial
Source: PLoS One. 2026 Jun 10;21(6):e0351060. doi: 10.1371/journal.pone.0351060 (PMC13252720; doi:10.1371/journal.pone.0351060)
Supplement: Attachment — Submitted filename: Reviewer comment.docx [file pone.0351060.s003.docx]

Dear Academic Editor,

We sincerely thank you and the reviewers for the careful evaluation of our manuscript entitled:

**“Effectiveness of Shortwave Diathermy in Patients with Chronic Low Back Pain: A Study Protocol for a Randomised, Single-Blinded, Multicentre Clinical Trial.”**

We are grateful for the constructive feedback, which has helped us improve the clarity, methodological rigor, and overall quality of the manuscript. We have revised the manuscript accordingly. All changes have been incorporated into the revised version, and key modifications are described below with approximate page and line references.

**Response to Reviewer #1**

**Comment 1:**

“Sample size calculation doesn’t correlation between repeated data points, which may lead to underpower.”

**Response:**
We thank the reviewer for this important observation. The sample size section has been revised (Methods, Sample-size calculation, Page ~11, Lines ~5–15) to clarify that the calculation is based on the primary outcome at the main follow-up time point. We have explicitly stated that repeated measurements will be analysed using linear mixed-effects models, which account for within-subject correlation and improve statistical efficiency. A conservative approach was adopted due to uncertainty in correlation parameters.

**Comment 2:**

“Missing data will be imputed with multiple imputations… What variables will be included for the imputation model?”

**Response:**
We have expanded the statistical analysis section (Page ~12–13, Lines ~20–30) to include full details of the imputation strategy. Missing data will be handled using Multiple Imputation by Chained Equations (MICE), assuming missing at random. The imputation model includes baseline outcomes, treatment allocation, and relevant demographic variables. At least 20 imputations will be generated, and all available data will be included without applying arbitrary missingness thresholds.

**Comment 3:**

“No plan for comparison between the people who complete and who don’t.”

**Response:**
We have added a statement in the Statistical Analysis section (Page ~13, Lines ~30–36) indicating that baseline characteristics of completers and non-completers will be compared to assess attrition bias. Additionally, a per-protocol analysis (≥80% adherence) has been included as a sensitivity analysis.

**Comment 4:**

“Details about intervention standardization should be provided.”

**Response:**
We have expanded the intervention section (Page ~12, Lines ~1–10) to include details on standardisation. All physiotherapists will undergo structured training using a treatment manual. Intervention fidelity will be ensured through monitoring, checklists, and supervision by the trial steering committee.

**Comment 5:**

“Please comment on the feasibility of recruiting the needed participants…”

**Response:**
We have added a recruitment feasibility statement (Trial status and timeline section, Page ~14, Lines ~10–15). Based on patient flow data (10–15 eligible participants per week per centre), recruitment of 208 participants across three centres is considered feasible within the planned timeframe.

**Comment 6:**

“Please provide clinical relevance and justification…”

**Response:**
The Introduction (Page ~5–6) and Discussion (Page ~15) have been strengthened to better highlight the clinical and health-system relevance, particularly in resource-constrained settings. The revised text emphasises the importance of evaluating the added value of shortwave diathermy and its implications for evidence-based physiotherapy practice.

**Response to Reviewer #2**

**Comment:**

“The study was conducted between January 2024 and July 2025. How was it a retrospective cohort study?”

**Response:**
We respectfully clarify that this manuscript is a **study protocol for a prospective randomised controlled trial**, not a retrospective cohort study. No participants have been recruited, and no data have been collected. This has been clarified in the Methods and Trial Status sections (Page ~14, Lines ~15–20).

**Comment:**

“The study design is confused…”

**Response:**
We have carefully revised the manuscript to ensure clarity. The study is clearly described as a multicentre, parallel-group, randomised, single-blind, sham-controlled trial (Methods, Study design, Page ~8).

**Comment:**

“This would not be rehabilitation…”

**Response:**
The intervention consists of a structured physiotherapy programme (exercise, education, and adjunct electrotherapy) delivered over four weeks, consistent with standard outpatient rehabilitation practice. This has been clarified in the Methods section (Page ~9–10).

**Comment:**

“Rehabilitation stays were 5.5 days…”

**Response:**
We respectfully clarify that this study does not involve inpatient rehabilitation. The intervention is outpatient-based. The manuscript has been revised to ensure clarity on this point.

**Comment:**

“The English language should be used properly.”

**Response:**
The manuscript has been thoroughly edited for grammar, clarity, and consistency throughout.

**Comment:**

“It could be a retrospective descriptive study.”

**Response:**
We reiterate that this is a prospective randomised controlled trial protocol. The study design has been clarified consistently throughout the manuscript.

**Response to Reviewer #3**

**Comment:**

“…electrotherapy… problem is its isolated use…”

**Response:**
We have revised the Introduction (Page ~5, Lines ~10–15) to reflect a balanced perspective. The text now states that electrotherapy can be beneficial as an adjunct, but its isolated use without concurrent exercise may limit effectiveness.

**Comment:**

“Suggest adding daily pain assessment…”

**Response:**
We thank the reviewer for this valuable suggestion. While daily pain assessment could provide additional insights, it was not included due to feasibility constraints and potential participant burden in a multicentre setting. This has been acknowledged as a potential direction for future research.

**Final Statement**

We sincerely thank the reviewers and the editor for their insightful comments. We believe that the revisions have significantly improved the manuscript and addressed all concerns raised. We hope the revised manuscript is now suitable for publication in *PLOS ONE*.

Sincerely,
**Dr. Mohammad Ali**
(On behalf of all authors)
