# Supplementary material for: Effectiveness of shortwave diathermy in patients with chronic low back pain: A study protocol for a randomised, single-blinded, multicentre clinical trial
Source: PLoS One. 2026 Jun 10;21(6):e0351060. doi: 10.1371/journal.pone.0351060 (PMC13252720; doi:10.1371/journal.pone.0351060)
Supplement: Peer review history

# Peer review: Decision Letter 0

**DOI:** 10.1371/journal.pone.0351060.r001

**Authors:** Md. Feroz Kabir

24 Mar 2026

PONE-D-25-62638

Effectiveness of Shortwave Diathermy in Patients with Chronic Low Back Pain: A Study Protocol for a Randomised, Single-Blinded, Multicentre Clinical Trial

PLOS One

Dear -->Dr. --> Ali,

Thank you for submitting your manuscript to PLOS ONE. After careful consideration, we feel that it has merit but does not fully meet PLOS ONE’s publication criteria as it currently stands. Therefore, we invite you to submit a revised version of the manuscript that addresses the points raised during the review process.

Please submit your revised manuscript by May 08 2026 11:59PM. If you will need more time than this to complete your revisions, please reply to this message or contact the journal office at plosone@plos.org. When you're ready to submit your revision, log on to https://www.editorialmanager.com/pone/ and select the 'Submissions Needing Revision' folder to locate your manuscript file.

Please include the following items when submitting your revised manuscript:

- A letter that responds to each point raised by the academic editor and reviewer(s). You should upload this letter as a separate file labeled 'Response to Reviewers'.
- A marked-up copy of your manuscript that highlights changes made to the original version. You should upload this as a separate file labeled 'Revised Manuscript with Track Changes'.
- An unmarked version of your revised paper without tracked changes. You should upload this as a separate file labeled 'Manuscript'.

If you would like to make changes to your financial disclosure, please include your updated statement in your cover letter. Guidelines for resubmitting your figure files are available below the reviewer comments at the end of this letter.

If applicable, we recommend that you deposit your laboratory protocols in protocols.io to enhance the reproducibility of your results. Protocols.io assigns your protocol its own identifier (DOI) so that it can be cited independently in the future. For instructions see: https://journals.plos.org/plosone/s/submission-guidelines#loc-laboratory-protocols. Additionally, PLOS ONE offers an option for publishing peer-reviewed Lab Protocol articles, which describe protocols hosted on protocols.io. Read more information on sharing protocols at https://plos.org/protocols?utm_medium=editorial-email&utm_source=authorletters&utm_campaign=protocols.

We look forward to receiving your revised manuscript.

Kind regards,

Md. Feroz Kabir, PhD, BPT, MPT, MPH, BPED, MPED

Academic Editor

PLOS One

Journal Requirements:

When submitting your revision, we need you to address these additional requirements.

1.Please ensure that your manuscript meets PLOS ONE's style requirements, including those for file naming. The PLOS ONE style templates can be found at

https://journals.plos.org/plosone/s/file?id=wjVg/PLOSOne_formatting_sample_main_body.pdf and

https://journals.plos.org/plosone/s/file?id=ba62/PLOSOne_formatting_sample_title_authors_affiliations.pdf

2. Please provide a complete Data Availability Statement in the submission form, ensuring you include all necessary access information or a reason for why you are unable to make your data freely accessible. If your research concerns only data provided within your submission, please write "All data are in the manuscript and/or supporting information files" as your Data Availability Statement.

3. When completing the data availability statement of the submission form, you indicated that you will make your data available on acceptance. We strongly recommend all authors decide on a data sharing plan before acceptance, as the process can be lengthy and hold up publication timelines. Please note that, though access restrictions are acceptable now, your entire data will need to be made freely accessible if your manuscript is accepted for publication. This policy applies to all data except where public deposition would breach compliance with the protocol approved by your research ethics board. If you are unable to adhere to our open data policy, please kindly revise your statement to explain your reasoning and we will seek the editor's input on an exemption. Please be assured that, once you have provided your new statement, the assessment of your exemption will not hold up the peer review process.

4. Please amend either the title on the online submission form (via Edit Submission) or the title in the manuscript so that they are identical.

5. Your ethics statement should only appear in the Methods section of your manuscript. If your ethics statement is written in any section besides the Methods, please move it to the Methods section and delete it from any other section. Please ensure that your ethics statement is included in your manuscript, as the ethics statement entered into the online submission form will not be published alongside your manuscript.

6. Please include captions for your Supporting Information files at the end of your manuscript, and update any in-text citations to match accordingly. Please see our Supporting Information guidelines for more information: http://journals.plos.org/plosone/s/supporting-information.

7. If the reviewer comments include a recommendation to cite specific previously published works, please review and evaluate these publications to determine whether they are relevant and should be cited. There is no requirement to cite these works unless the editor has indicated otherwise.

Additional Editor Comments:

Please submit the revised manuscript within the next 3 weeks.

[Note: HTML markup is below. Please do not edit.]

Reviewer's Responses to Questions

**Comments to the Author**

1. Does the manuscript provide a valid rationale for the proposed study, with clearly identified and justified research questions?

The research question outlined is expected to address a valid academic problem or topic and contribute to the base of knowledge in the field.

Reviewer #1: Partly

Reviewer #2: No

Reviewer #3: Yes

**********

2. Is the protocol technically sound and planned in a manner that will lead to a meaningful outcome and allow testing the stated hypotheses?

The manuscript should describe the methods in sufficient detail to prevent undisclosed flexibility in the experimental procedure or analysis pipeline, including sufficient outcome-neutral conditions (e.g. necessary controls, absence of floor or ceiling effects) to test the proposed hypotheses and a statistical power analysis where applicable. As there may be aspects of the methodology and analysis which can only be refined once the work is undertaken, authors should outline potential assumptions and explicitly describe what aspects of the proposed analyses, if any, are exploratory.

Reviewer #1: Yes

Reviewer #2: No

Reviewer #3: Yes

**********

3. Is the methodology feasible and described in sufficient detail to allow the work to be replicable?

Descriptions of methods and materials in the protocol should be reported in sufficient detail for another researcher to reproduce all experiments and analyses. The protocol should describe the appropriate controls, sample size calculations, and replication needed to ensure that the data are robust and reproducible.

Reviewer #1: Yes

Reviewer #2: No

Reviewer #3: Yes

**********

4. Have the authors described where all data underlying the findings will be made available when the study is complete?

The PLOS Data policy requires authors to make all data underlying the findings described in their manuscript fully available without restriction, with rare exception, at the time of publication. The data should be provided as part of the manuscript or its supporting information, or deposited to a public repository. For example, in addition to summary statistics, the data points behind means, medians and variance measures should be available. If there are restrictions on publicly sharing data—e.g. participant privacy or use of data from a third party—those must be specified.

Reviewer #1: Yes

Reviewer #2: Yes

Reviewer #3: Yes

**********

5. Is the manuscript presented in an intelligible fashion and written in standard English?

PLOS ONE does not copyedit accepted manuscripts, so the language in submitted articles must be clear, correct, and unambiguous. Any typographical or grammatical errors should be corrected at revision, so please note any specific errors here.

Reviewer #1: Yes

Reviewer #2: Yes

Reviewer #3: Yes

**********

6. Review Comments to the Author

Please use the space provided to explain your answers to the questions above and, if applicable, provide comments about issues authors must address before this protocol can be accepted for publication. You may also include additional comments for the author, including concerns about research or publication ethics.

You may also provide optional suggestions and comments to authors that they might find helpful in planning their study.

(Please upload your review as an attachment if it exceeds 20,000 characters)

Reviewer #1: The authors intended to recruit 208 participants with CLBP who will be 1:1 randomized to receive CSWD or sham CSWD to evaluate whether CSWD combined with standard physiotherapy provides additional benefits beyond sham CSWD with physiotherapy.

1. Sample size calculation doesn’t correlation between repeated data points, which may lead to underpower.

2. Missing data will be imputed with multiple imputations. However, there is no information on the details. For example, will all the imputation, no matter how many missingness, be imputed? What variables will be included for the imputation model?

3. The study team assumes 80% adherence rate. However, there is no plan for comparison between the people who complete and who don’t.

4. Details about intervention standardization should be provided.

5. Please comment on the feasibility of recruiting the needed participants within the planned timeframe.

6. Please provide clinical relevance and justification of the planned work.

Reviewer #2: The study was conducted between January 2024 and July 2025. How was it a retrospective cohort study? It is very immediate past.

The study design is confused, and it should be rectified properly.

This would not be rehabilitation, which is the title, because rehabilitation would have a protocol and require a minimum number of days.

The rehabilitation services were not addressed according to the Rehabilitation protocol.

Rehabilitation stays were 5.5 days. Is it possible to rehabilitate within this time?

The screening procedure was systematic.

The English language should be used properly.

It could be a retrospective descriptive study.

Reviewer #3: I consider it very important to conduct a study of this nature addressing this topic, as there is a need for good clinical trials using electrophysical agents.

“ Physiotherapy services are underutilised and often influenced by outdated or passive treatment paradigms that rely heavily on electrotherapy rather than active, exercise-based interventions [5].”

This section needs to be modified. Electrotherapy, as an adjunct to treatment, has shown many benefits. The problem is its isolated use. Therefore, I suggest rewriting the text to point out that the combination of electrophysical agents with exercise is not used in many therapeutic approaches.

Looking at studies with electrophysical agents in general, one unanswered question is the effectiveness of these agents throughout the treatment. If it were possible to assess pain daily using a numerical pain scale by patients, this study could make a significant contribution to understanding when they should be used during treatment. It may be that their effect is very good in the first 10 days of treatment and then no longer necessary. Exercise alone would then be effective. Therefore, I suggest adding daily pain assessment to the research protocol, which could greatly contribute to the use of electrophysical agents in the treatment of musculoskeletal dysfunctions. This important information is not yet discussed in any article addressing this topic.

**********

7. PLOS authors have the option to publish the peer review history of their article (what does this mean?). If published, this will include your full peer review and any attached files.

If you choose “no”, your identity will remain anonymous but your review may still be made public.

**Do you want your identity to be public for this peer review?** For information about this choice, including consent withdrawal, please see our Privacy Policy.

Reviewer #1: No

Reviewer #2: **Yes:** Dr. Md. Shafiullah Prodhania

Reviewer #3: No

**********

[NOTE: If reviewer comments were submitted as an attachment file, they will be attached to this email and accessible via the submission site. Please log into your account, locate the manuscript record, and check for the action link "View Attachments". If this link does not appear, there are no attachment files.]

To ensure your figures meet our technical requirements, please review our figure guidelines: https://journals.plos.org/plosone/s/figures

You may also use PLOS’s free figure tool, NAAS, to help you prepare publication quality figures: https://journals.plos.org/plosone/s/figures#loc-tools-for-figure-preparation.

NAAS will assess whether your figures meet our technical requirements by comparing each figure against our figure specifications.

# Author comment: Author response to Decision Letter 1

**DOI:** 10.1371/journal.pone.0351060.r002

12 May 2026

Response to Reviewer #1

________________________________________

Comment 1:

“Sample size calculation doesn’t correlation between repeated data points, which may lead to underpower.”

Response:

We thank the reviewer for this important observation. The sample size section has been revised (Methods, Sample-size calculation, Page ~11, Lines ~5–15) to clarify that the calculation is based on the primary outcome at the main follow-up time point. We have explicitly stated that repeated measurements will be analysed using linear mixed-effects models, which account for within-subject correlation and improve statistical efficiency. A conservative approach was adopted due to uncertainty in correlation parameters.

________________________________________

Comment 2:

“Missing data will be imputed with multiple imputations… What variables will be included for the imputation model?”

Response:

We have expanded the statistical analysis section (Page ~12–13, Lines ~20–30) to include full details of the imputation strategy. Missing data will be handled using Multiple Imputation by Chained Equations (MICE), assuming missing at random. The imputation model includes baseline outcomes, treatment allocation, and relevant demographic variables. At least 20 imputations will be generated, and all available data will be included without applying arbitrary missingness thresholds.

________________________________________

Comment 3:

“No plan for comparison between the people who complete and who don’t.”

Response:

We have added a statement in the Statistical Analysis section (Page ~13, Lines ~30–36) indicating that baseline characteristics of completers and non-completers will be compared to assess attrition bias. Additionally, a per-protocol analysis (≥80% adherence) has been included as a sensitivity analysis.

________________________________________

Comment 4:

“Details about intervention standardization should be provided.”

Response:

We have expanded the intervention section (Page ~12, Lines ~1–10) to include details on standardisation. All physiotherapists will undergo structured training using a treatment manual. Intervention fidelity will be ensured through monitoring, checklists, and supervision by the trial steering committee.

________________________________________

Comment 5:

“Please comment on the feasibility of recruiting the needed participants…”

Response:

We have added a recruitment feasibility statement (Trial status and timeline section, Page ~14, Lines ~10–15). Based on patient flow data (10–15 eligible participants per week per centre), recruitment of 208 participants across three centres is considered feasible within the planned timeframe.

________________________________________

Comment 6:

“Please provide clinical relevance and justification…”

Response:

The Introduction (Page ~5–6) and Discussion (Page ~15) have been strengthened to better highlight the clinical and health-system relevance, particularly in resource-constrained settings. The revised text emphasises the importance of evaluating the added value of shortwave diathermy and its implications for evidence-based physiotherapy practice.

________________________________________

Response to Reviewer #2

________________________________________

Comment:

“The study was conducted between January 2024 and July 2025. How was it a retrospective cohort study?”

Response:

We respectfully clarify that this manuscript is a study protocol for a prospective randomised controlled trial, not a retrospective cohort study. No participants have been recruited, and no data have been collected. This has been clarified in the Methods and Trial Status sections (Page ~14, Lines ~15–20).

________________________________________

Comment:

“The study design is confused…”

Response:

We have carefully revised the manuscript to ensure clarity. The study is clearly described as a multicentre, parallel-group, randomised, single-blind, sham-controlled trial (Methods, Study design, Page ~8).

________________________________________

Comment:

“This would not be rehabilitation…”

Response:

The intervention consists of a structured physiotherapy programme (exercise, education, and adjunct electrotherapy) delivered over four weeks, consistent with standard outpatient rehabilitation practice. This has been clarified in the Methods section (Page ~9–10).

________________________________________

Comment:

“Rehabilitation stays were 5.5 days…”

Response:

We respectfully clarify that this study does not involve inpatient rehabilitation. The intervention is outpatient-based. The manuscript has been revised to ensure clarity on this point.

________________________________________

Comment:

“The English language should be used properly.”

Response:

The manuscript has been thoroughly edited for grammar, clarity, and consistency throughout.

________________________________________

Comment:

“It could be a retrospective descriptive study.”

Response:

We reiterate that this is a prospective randomised controlled trial protocol. The study design has been clarified consistently throughout the manuscript.

________________________________________

Response to Reviewer #3

________________________________________

Comment:

“…electrotherapy… problem is its isolated use…”

Response:

We have revised the Introduction (Page ~5, Lines ~10–15) to reflect a balanced perspective. The text now states that electrotherapy can be beneficial as an adjunct, but its isolated use without concurrent exercise may limit effectiveness.

________________________________________

Comment:

“Suggest adding daily pain assessment…”

Response:

We thank the reviewer for this valuable suggestion. While daily pain assessment could provide additional insights, it was not included due to feasibility constraints and potential participant burden in a multicentre setting. This has been acknowledged as a potential direction for future research.

________________________________________

Attachment Submitted filename: Reviewer comment.docx

# Peer review: Decision Letter 1

**DOI:** 10.1371/journal.pone.0351060.r003

**Authors:** Md. Feroz Kabir

22 May 2026

Effectiveness of Shortwave Diathermy in Patients with Chronic Low Back Pain: A Study Protocol for a Randomised, Single-Blinded, Multicentre Clinical Trial

PONE-D-25-62638R1

Dear Mohammad Ali,

We’re pleased to inform you that your manuscript has been judged scientifically suitable for publication and will be formally accepted for publication once it meets all outstanding technical requirements.

Within one week, you’ll receive an e-mail detailing the required amendments. When these have been addressed, you’ll receive a formal acceptance letter and your manuscript will be scheduled for publication.

An invoice will be generated when your article is formally accepted. Please note, if your institution has a publishing partnership with PLOS and your article meets the relevant criteria, all or part of your publication costs will be covered. Please make sure your user information is up-to-date by logging into Editorial Manager at Editorial Manager® and clicking the ‘Update My Information' link at the top of the page. For questions related to billing, please contact billing support.

If your institution or institutions have a press office, please notify them about your upcoming paper to help maximize its impact. If they’ll be preparing press materials, please inform our press team as soon as possible -- no later than 48 hours after receiving the formal acceptance. Your manuscript will remain under strict press embargo until 2 pm Eastern Time on the date of publication. For more information, please contact onepress@plos.org.

Kind regards,

Md. Feroz Kabir, PhD, BPT, MPT, MPH, BPED, MPED

Academic Editor

PLOS One

Additional Editor Comments (optional):

Please correct the english language and formatting properly.

Reviewers' comments:

Reviewer's Responses to Questions

**Comments to the Author**

1. Does the manuscript provide a valid rationale for the proposed study, with clearly identified and justified research questions?

The research question outlined is expected to address a valid academic problem or topic and contribute to the base of knowledge in the field.

Reviewer #1: Yes

**********

2. Is the protocol technically sound and planned in a manner that will lead to a meaningful outcome and allow testing the stated hypotheses?

The manuscript should describe the methods in sufficient detail to prevent undisclosed flexibility in the experimental procedure or analysis pipeline, including sufficient outcome-neutral conditions (e.g. necessary controls, absence of floor or ceiling effects) to test the proposed hypotheses and a statistical power analysis where applicable. As there may be aspects of the methodology and analysis which can only be refined once the work is undertaken, authors should outline potential assumptions and explicitly describe what aspects of the proposed analyses, if any, are exploratory.

Reviewer #1: Yes

**********

3. Is the methodology feasible and described in sufficient detail to allow the work to be replicable?

Descriptions of methods and materials in the protocol should be reported in sufficient detail for another researcher to reproduce all experiments and analyses. The protocol should describe the appropriate controls, sample size calculations, and replication needed to ensure that the data are robust and reproducible.

Reviewer #1: Yes

**********

4. Have the authors described where all data underlying the findings will be made available when the study is complete?

The PLOS Data policy requires authors to make all data underlying the findings described in their manuscript fully available without restriction, with rare exception, at the time of publication. The data should be provided as part of the manuscript or its supporting information, or deposited to a public repository. For example, in addition to summary statistics, the data points behind means, medians and variance measures should be available. If there are restrictions on publicly sharing data—e.g. participant privacy or use of data from a third party—those must be specified.

Reviewer #1: Yes

**********

5. Is the manuscript presented in an intelligible fashion and written in standard English?

PLOS ONE does not copyedit accepted manuscripts, so the language in submitted articles must be clear, correct, and unambiguous. Any typographical or grammatical errors should be corrected at revision, so please note any specific errors here.

Reviewer #1: Yes

**********

6. Review Comments to the Author

Please use the space provided to explain your answers to the questions above and, if applicable, provide comments about issues authors must address before this protocol can be accepted for publication. You may also include additional comments for the author, including concerns about research or publication ethics.

You may also provide optional suggestions and comments to authors that they might find helpful in planning their study.

(Please upload your review as an attachment if it exceeds 20,000 characters)

Reviewer #1: Thanks for satisfactorily addressing all the raised comments. This reviewer has no more further concerns.

**********

7. PLOS authors have the option to publish the peer review history of their article (what does this mean?). If published, this will include your full peer review and any attached files.

If you choose “no”, your identity will remain anonymous but your review may still be made public.

**Do you want your identity to be public for this peer review?** For information about this choice, including consent withdrawal, please see our Privacy Policy.

Reviewer #1: No

**********

# Editor report: Acceptance letter

**DOI:** 10.1371/journal.pone.0351060.r004

**Authors:** Md. Feroz Kabir

PONE-D-25-62638R1

PLOS One

Dear Dr. Ali,

I'm pleased to inform you that your manuscript has been deemed suitable for publication in PLOS One. Congratulations! Your manuscript is now being handed over to our production team.

At this stage, our production department will prepare your paper for publication. This includes ensuring the following:

* All references, tables, and figures are properly cited

* All relevant supporting information is included in the manuscript submission,

* There are no issues that prevent the paper from being properly typeset

You will receive further instructions from the production team, including instructions on how to review your proof when it is ready. Please keep in mind that we are working through a large volume of accepted articles, so please give us a few days to review your paper and let you know the next and final steps.

Lastly, if your institution or institutions have a press office, please let them know about your upcoming paper now to help maximize its impact. If they'll be preparing press materials, please inform our press team within the next 48 hours. Your manuscript will remain under strict press embargo until 2 pm Eastern Time on the date of publication. For more information, please contact onepress@plos.org.

You will receive an invoice from PLOS for your publication fee after your manuscript has reached the completed accept phase. If you receive an email requesting payment before acceptance or for any other service, this may be a phishing scheme. Learn how to identify phishing emails and protect your accounts at https://explore.plos.org/phishing.

If we can help with anything else, please email us at customercare@plos.org.

Thank you for submitting your work to PLOS ONE and supporting open access.

Kind regards,

PLOS ONE Editorial Office Staff

on behalf of

Dr. Md. Feroz Kabir

Academic Editor

PLOS One
